# Surgical therapy of recurrent genital cysts

**DOI:** 10.1111/ddg.15863

**Published:** 2025-09-14

**Authors:** Julia Sipos, Jörg Neymeyer, Veronika Weger, Sylke Schneider‐Burrus

**Affiliations:** ^1^ Zentrum für Dermatochirurgie Havelklinik Berlin; ^2^ Klinik für Urologie Charité – Universitätsmedizin Berlin; ^3^ MVZ im Fürstenberg‐Karree, Berlin; ^4^ Translational Skin Inflammation Research Department of Dermatology, Venereology and Allergology Charité – Universitätsmedizin Berlin

**Keywords:** epidermal testicular cyst, genital cysts, sebaceous cyst of the scrotum, scrotal cyst, vulvar cyst

## INTRODUCTION

Genital cysts are rare lesions that develop in increasing numbers and sizes, primarily on the scrotum or vulva, and may calcify over time.^1^ Their prognosis is generally favorable, as malignant transformation is very rare. However, secondary infections have been reported that can lead to Fournier gangrene and sepsis.^2^ Conservative treatments for genital cysts appear to be ineffective.^3^ Therefore, surgical methods such as pinch‐punch incision and extirpation of individual cysts are almost exclusively used.^4^ The drawback of this method is a high recurrence rate.^5^

We report here on three patients in whom wide excision of the cyst‐covered genital skin followed by reconstruction of the scrotum or labia led to cosmetically good results and long‐term therapeutic success.

## TECHNIQUE

Three patients aged 23, 25, and 57 presented with numerous recurrent cysts in the scrotal region or on the labia majora.

**FIGURE 1:**
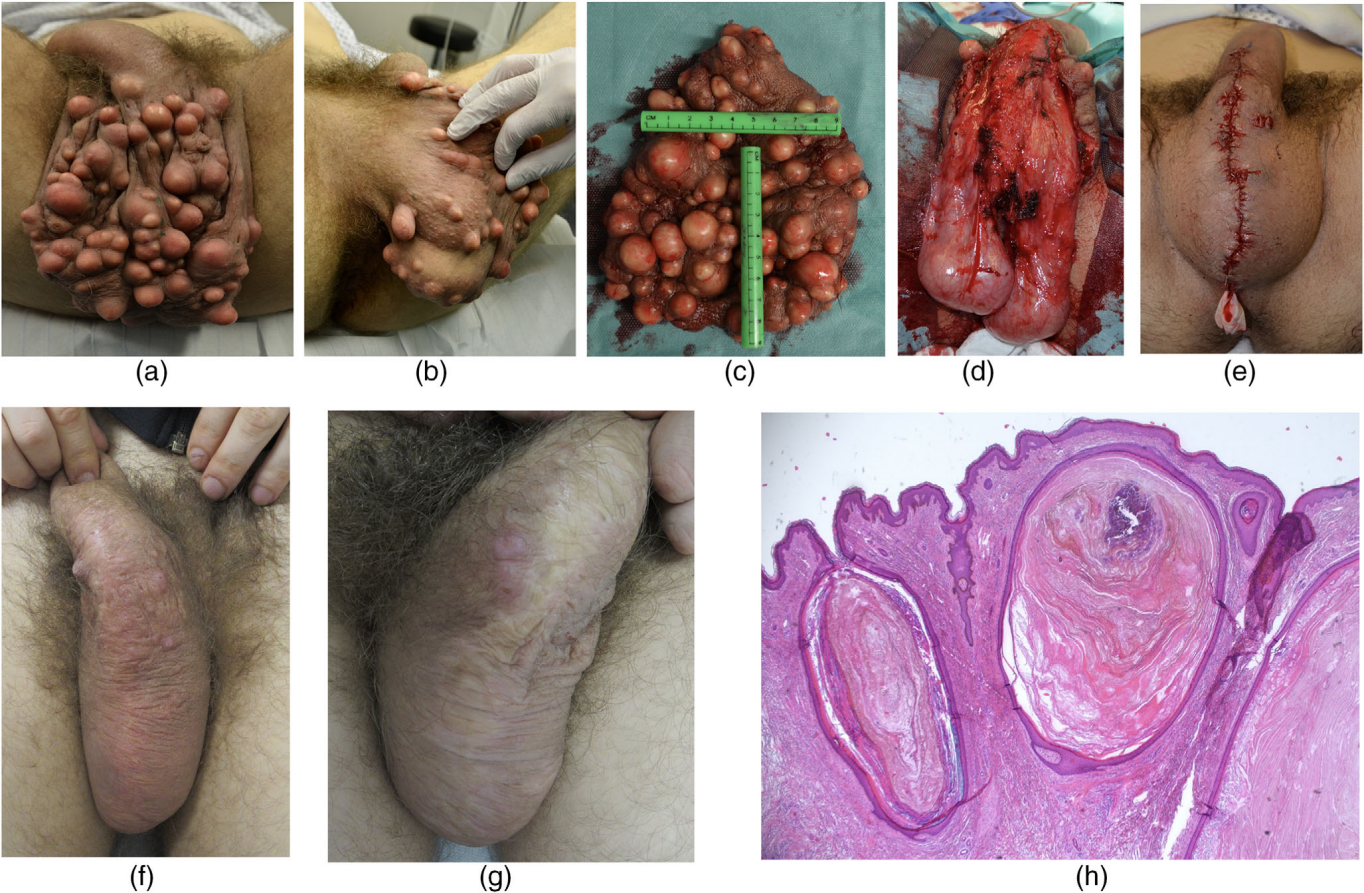
(a) Twenty‐two‐year‐old male patient with over 200 tense, non‐inflammatory cysts on the scrotum, measuring between 0.5 and 3 cm in diameter, extending to the penile shaft. (b) Cysts predominantly located medially, with minute satellite cysts. (c) Resected scrotal skin specimen, measuring 12 × 18 cm. (d) Intraoperative view after extensive resection of the scrotal skin. (e) Postoperative result following cyst removal and scrotoplasty; all visible cysts excised, scrotum markedly reduced in size. (f) Clinical result at 8 months postoperatively. (g) Clinical result at 19 months postoperatively. (h) Histological section (hematoxylin‐eosin stain, original magnification × 4) showing large cysts and minute cysts less than 1 mm in diameter.

Patient 1, aged 22, presented again after a total of seven surgical procedures for scrotal epidermoid cysts, the last of which had been four years ago. The examination revealed over 200 cysts on the scrotum with a diameter of up to 3 cm (Figures 1a,b). The cysts were located medially up to about 4 cm on both sides of the raphe scroti. In addition, a significant elongation of the entire scrotum was observed.

**FIGURE 2:**
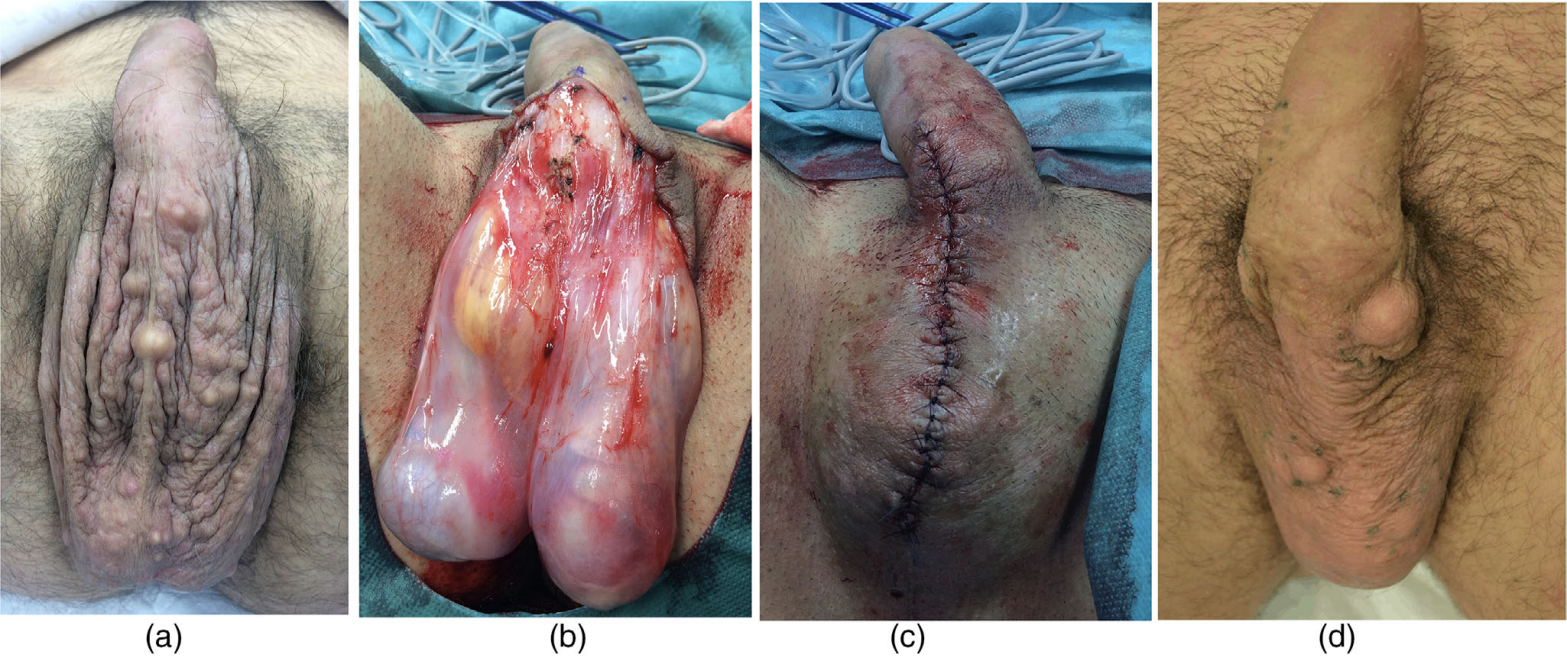
(a) Twenty‐five‐year‐old male patient, preoperative condition with numerous cysts across the entire scrotum. (b) Intraoperative view after scrotal skin resection. (c) Postoperative result following scrotoplasty. (d) Clinical result at 42 months postoperatively, showing localized recurrence with isolated cysts.

Patient 2, 25 years old, presented with scrotal cysts that had been present since puberty. Multiple scrotal cysts had been excised 7, 6, and 4 years ago. Upon presentation at our clinic in November 2018 (Figure 2a), multiple scrotal nodules were visible, some of which were confluent, movable in relation to the testicles with a diameter of up to 1.5 cm. Most of the nodules were located medially in close proximity to the raphe scroti. The scrotum showed a coarsening of the skin folds and an excess of skin unusual for his young age.

**FIGURE 3:**
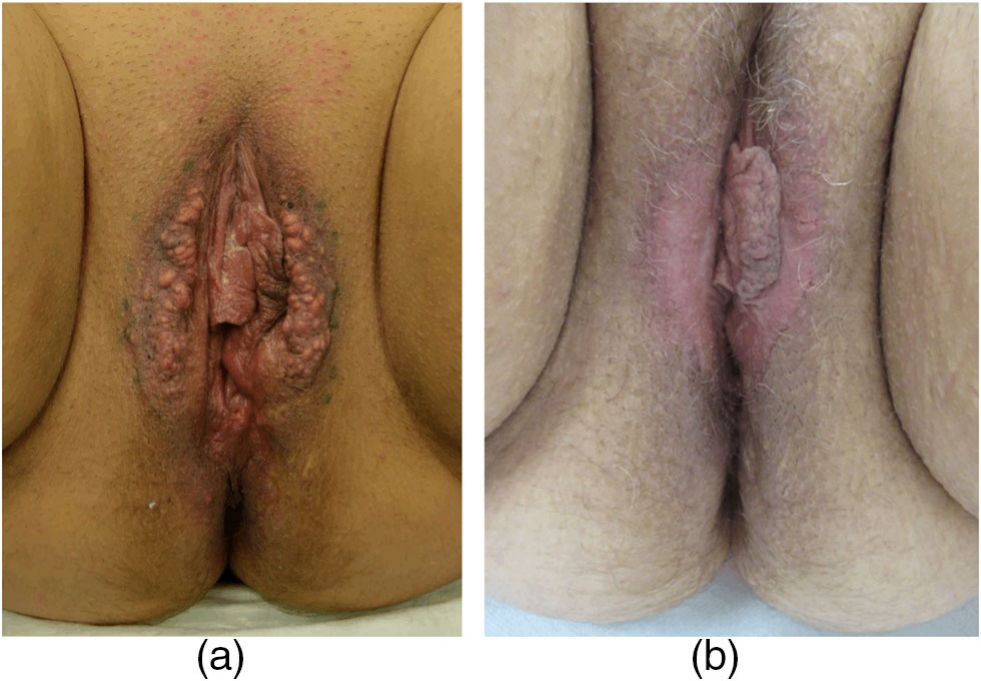
(a) Preoperative condition of a 57‐year‐old female patient with numerous cysts of the labia majora; the labia minora are unaffected. (b) Postoperative result showing cyst‐free and symmetric labia.

Patient 3, 57 years old, had suffered from multiple cysts on the labia majora since puberty. No surgical treatment had been performed to date. On examination, approximately 150 partially open and partially closed cysts with a diameter of 1 to 10 mm were found on the labia majora (Figure 3a). In addition, there was a sac‐like excess of genital skin.

In all three patients, the medial two‐thirds of the scrotal skin or the skin of the labia majora was excised (Figures 1c, d; 2b), followed by reconstruction using resorbable, epicutaneous, sutures (Figures 1, 2). Over the next 3 years, patient 2 returned for excision of individual cysts (Figure 2d). No relapses occurred in patients 1 and 3 (Figure 1f, g; 3b).

## COMMENTS

Due to their number and size, genital cysts can lead to genital mutilation. Other chronic skin diseases are known to have a negative impact on patients' sexual health, particularly when they affect the genital area, and are associated with anxiety disorders and increased depression.^6^, ^7^, ^8^

Treatment options for genital cysts are limited to surgical procedures such as pinch‐punch excision of individual cysts or the use of a CO_2_ laser to open and then extirpate the cysts.^4^, ^8^ The drawback of these treatment methods is the recurrence rate: Local relapses develop from tiny cysts that are easily overlooked during surgery (Figure 1h).^4^ In the two male patients presented in this paper, extensive recurrences were repeatedly observed after pinch‐punch biopsies of cysts.

We describe a treatment method in which the skin of the scrotum or labia majora is subtotally resected and surface continuity restored utilizing the skin remaining on the lateral side. This is a relatively simple and rapid procedure, as the cysts are mostly superficially located. ^9^ The deeper scrotal skin does not need to be exposed. Due to the size of the resected areas, the procedures were performed on an inpatient basis using a combination of total intravenous anesthesia and tumescent anesthesia. As a result of the surgical procedure, the preoperatively significantly lax scrotal skin and the skin of the labia majora were tightened, which was perceived as cosmetically favorable by the patients. Due to the considerable reservoir function of the heavily wrinkled skin of the scrotum and labia majora, it is possible to resect even large areas of skin without causing functional impairment.^10^, ^11^, ^12^

For spermatogenesis, it is important that the temperature of the testicles is about 2–8°C lower than the core body temperature.^12^ Therefore, no more than 70% of the skin of the scrotum should be resected to prevent displacement of the testicles, ensure adequate cooling, and avoid impairment of spermatogenesis.^4^, ^10^, ^11^, ^12^

Over the course of 3–5 years, patient 2 returned for resection of individual cysts on otherwise unremarkable scrotal skin; none of the patients experienced a fulminant relapse of the disease. Similar results were reported by Noel et al., who observed no new lesions during a one‐year follow‐up period after subtotal excision of scrotal skin.^13^

The pros and cons of the procedure should be discussed with the patient in detail before surgery: The procedure is associated with an increased risk of bleeding; in addition, patients should be informed about the possibility of impaired spermatogenesis after surgery. If family planning is not complete, cryopreservation is recommended.

In the authors' opinion, the good cosmetic result in an otherwise mutilating disease justifies this more elaborate procedure.

## CONFLICT OF INTEREST STATEMENT

None.
